# Simple Fast Quantification of Cholecalciferol, 25-Hydroxyvitamin D and 1,25-Dihydroxyvitamin D in Adipose Tissue Using LC-HRMS/MS

**DOI:** 10.3390/nu11091977

**Published:** 2019-08-22

**Authors:** Laurianne Bonnet, Marielle Margier, Ljubica Svilar, Charlene Couturier, Emmanuelle Reboul, Jean-Charles Martin, Jean-François Landrier, Catherine Defoort

**Affiliations:** 1 C2VN (Centre de Recherche CardioVasculaire et Nutrition), INRA, INSERM, Université d’Aix-Marseille, Faculté de Médecine, 27 Bd Jean Moulin, 13385 Marseille CEDEX 05, France; 2 CriBioM, Criblage Biologique Marseille, Faculté de médecine de la Timone, 13005 Marseille, France

**Keywords:** vitamin D metabolite quantification, LC-MS/MS, adipose tissue, obesity

## Abstract

Vitamin D metabolism is actively modulated in adipose tissue during obesity. To better investigate this process, we develop a specific LC-HRMS/MS method that can simultaneously quantify three vitamin D metabolites, i.e., cholecalciferol, 25-hydroxyvitamin D_3_ (25(OH)D_3_), and 1,25-dihydroxyvitamin D_3_ (1,25(OH)_2_D_3_) in a complex matrix, such as mouse adipose tissue and plasma. The method uses pretreatment with liquid–liquid or solid–phase extraction followed by derivatization using Amplifex^®^ reagents to improve metabolite stability and ionization efficiency. Here, the method is optimized by co-eluting stable isotope-labelled internal standards to calibrate each analogue and to spike biological samples. Intra-day and inter-day relative standard deviations were 0.8–6.0% and 2.0–14.4%, respectively for the three derivatized metabolites. The limits of quantification (LoQ) achieved with Amplifex^®^ derivatization were 0.02 ng/mL, 0.19 ng/mL, and 0.78 ng/mL for 1,25(OH)_2_D_3_, 25(OH)D_3_ and cholecalciferol, respectively. Now, for the first time, 1,25(OH)_2_D_3_ can be co-quantified with cholecalciferol and 25(OH)D_3_ in mouse adipose tissue. This validated method is successfully applied to study the impact of obesity on vitamin D status in mice.

## 1. Introduction

Vitamin D (VD), or cholecalciferol, is a prohormone involved in calcium and phosphate homeostasis. VD insufficiency, or deficiency, is directly linked to well-known bone diseases including rickets, osteoporosis, and osteomalacia [1], and may be involved in a number of other pathophysiological disorders, including cardiovascular disease or type 2 diabetes. After endogenous synthesis or intestinal absorption [2], cholecalciferol is metabolized in the liver by 25-hydroxylases that produce 25-hydroxyvitamin D [3]. The major form of VD found in plasma is 25(OH)D_3_, which is considered the best biomarker of VD status [4]. 25(OH)D can be hydroxylated in the kidney or in target tissues [5] by CYP27B1 into 1α,25-dihydroxyvitamin D, which is the active form of VD. 

Several studies and a recent review [6,7] highlighted that adipose tissue is one of the main storage sites for cholecalciferol and 25(OH)D3, and the mechanism of cholecalciferol and 25(OH)D_3_ uptake by adipocytes was recently identified [8]. Obesity is commonly associated with vitamin D deficiency [9,10], but there is no clear picture or even consensus on the mechanism(s) underpinning vitamin D status in obesity. One often-cited mechanism is deposition and sequestration of vitamin D metabolites in adipose tissue stores, but there is little experimental evidence to support this hypothesis. We recently demonstrated that cholecalciferol and 25(OH)D3 storage could result from an active mechanism involving modulated expression of several cytochromes [11].

One of the most informative ways to explore VD-related health and metabolism is to simultaneously measure VD metabolites in key organs. Consequently, the frequency of routine analytical measurement of VD status has increased greatly [12], necessitating the development of reliable and accurate analytical methods to measure concentration levels of VD metabolites not only in plasma but also in relevant body tissues [13]. Numerous liquid chromatography-tandem mass spectrometry (LC-MS/MS) methods have been described for measuring VD metabolites individually or in combination [14,15,16,17,18,19,20,21], but few papers reported the absolute quantification of cholecalciferol and 25(OH)D3 in adipose tissue by high-performance liquid chromatography tandem-mass spectrometry (HPLC-MS/MS) [22,23,24], and none of them include the detection of 1,25(OH)2D3 with satisfactory sensitivity [25,26]. 

Here, to address this gap, we set out to develop and validate a liquid-chromatography high-resolution tandem mass spectrometry (LC-HRMS/MS) method for simultaneous and accurate determination of cholecalciferol, 25(OH)D3 and 1,25(OH)2D3 in mouse adipose tissue. This effort carried a number of challenges, chiefly (i) the low in-sample concentrations of these metabolites, especially 1,25(OH)2D3 which is biologically active at picomolar concentrations, and (ii) the small amounts of tissue samples available, especially samples from mouse studies. The need to increase ionization efficiency and sensitivity implied a sample pretreatment, so we opt to use Amplifex^®^ as a derivatizing reagent applicable to diene analytes. We apply our validated method to quantify the three vitamin D metabolites in adipose tissue and plasma from C57BL/6J mice fed for 11 weeks on either a control diet (control, 10% energy from fat) or a high-fat diet (HF, 60% energy from fat) formulated to provide equivalent vitamin D intake in both groups.

## 2. Material and Methods

### 2.1. Materials

Cholecalciferol, 25(OH)D3, 1,25(OH)2D3, dibasic potassium phosphate (K_2_HPO_4_), methyl tert-butyl ether (MTBE), and phosphate buffer saline (PBS) were purchased from Sigma–Aldrich. Amplifex^®^ Diene reagent was purchased from Sciex (Chemistry and Consumables R&D, Framingham, MA). Isotopically-labelled d3-cholecalciferol, d3-25(OH)D3, and d3-1,25(OH)2D3 were purchased from Cambridge Isotope Laboratories (MA). These three labelled molecules were deuterated in position 6,19,19 and have been used as internal standards for the precision of the method. All vitamin D metabolites were stored at −80 °C in the dark. LC-MS-grade acetonitrile (AcN) and formic acid and HPLC-grade methanol (MeOH) and ethyl acetate (EtOAc) were obtained from Carlo Erba Reagents (Peypin, France). The water used was generated through a Direct-Q Ultrapure Water System from Millipore (Bedford, MA) with a specific resistance of 18.2 MΩ cm. Oasis HLB cartridges used for solid-phase extraction (3 CC/60 mg) were supplied by Waters (Guyancourt, France). Mouse samples were taken on euthanized animals (C57Bl/6J male mice) according to the local animal ethics committee protocols (APAFIS#2595-2016091911217758).

### 2.2. Instrumentation

Chromatographic separation was carried out on a Dionex UltiMate 3000 system (Thermo Fisher Scientific, Waltham, MA) configured with a rapid separation (RS) pump (LPG-3400 RS), an autosampler (WPS-3000 TRS) and a column compartment (TCC-3000 RS), all operated by Chromeleon 6.8 software. Samples were injected on a Hypersil GOLD C18 column (2.1 × 100 mm; Thermo Scientific, Les Ulis, France). Parallel reaction monitoring (PRM and accurate mass measurements were performed on a Q-Exactive Plus mass spectrometer (Thermo Fisher Scientific, Bremen, Germany) with a heated electrospray ionization (H-ESI II) probe. Thermo Xcalibur 3.0.63 software was used for both instrument setup and LC-MS system control during data acquisition, and for data treatment. Q Exactive Plus Tune 2.5 software was used for direct control of the mass spectrometer. 

### 2.3. Chromatography Conditions 

The autosampler tray was kept at 4 °C during the experiment, and column oven temperature was regulated at 40 °C. 5 µL of sample was injected onto the column. Mobile phase A consisted of 0.1% formic acid in water, and mobile phase B was 0.1% formic acid in AcN. Solvent flow rate was set to 400 µL/min. Starting mobile phase consisted of 30% of solvent B held for 4 min. A linear gradient was applied with B % increased to 65% for 6 min, held isocratic for 2 min, then increased to 100% of B for 4 min. Start conditions were reinstated in 2 min, making the total run time 18 min.

### 2.4. Mass Spectrometry Conditions

LC-HRMS/MS analyses were performed with external calibration in positive mode providing a mass precision lower than 5 ppm. The H-ESI probe was kept at 310 °C. Transfer capillary temperature was kept at 320 °C. Spray voltage was set at 3500 V and the S-lens RF level was set at 55 V. Sheath and auxiliary gas were held at 30 and 8 (arbitrary units). 

PRM was set up in MS/MS acquisition mode, and full higher-energy collision dissociation (HCD) spectra were acquired. For each compound, the most abundant ion was selected as precursor ion and isolated in a 2 Uma window in a specified time segment and fragmented under previously optimized collision energies (Table 1). Resolving power was set to 35,000 full width at half maximum (FWMH) at *m*/*z* 200 and the Auto Gain Control (AGC) was set to 2 × 10^5^. Injection time was set to a maximum of 100 ms in order to optimize the analytical cycle time. 

### 2.5. Preparation of stock and working solutions of analytical and deuterated standards (IS)

A working solution of deuterated analytes was prepared at 0.02 ng/mL of each internal standard (IS) (d3-cholecalciferol, d3-25(OH)D3 and d3-1,25(OH)2D3) in ethanol. Stock solutions of cholecalciferol, 25(OH)D3 and 1,25(OH)2D3 standards were prepared at 100, 50 and 10 ng/mL, respectively in ethanol and stored at −80 °C in the dark. Calibration curves were prepared by serial dilution of the three analyte stock solutions to obtain calibration standards from 0 to 50 ng/mL. After 1.5 µL of the working solution of deuterated analytes was added to each dilution, derivatization was performed using Amplifex^®^. A batch of Quality Control (QC) samples was produced for method validation.

### 2.6. Animals, Diets, and Experiments

Six-week-old male C57BL/6J mice were obtained from Janvier Labs (Le Genest-Saint-Isle, France) and fed ad libitum with standard chow (maintenance diet A04, Safe Diets, Augy, France) during a one week acclimatization period with ad libitum access to drinking water, housed at 22 °C under a 12 h/12 h light/dark cycle at 20% relative humidity. The mice were then divided into two groups: a control-diet group (control: 10% energy from lipids, *n* = 10) and a high-fat-diet group (HF: 60% energy from lipids, *n* = 10) (TestDiet, London, UK). Blood and adipose tissue were collected after 11 weeks on either the control or high-fat diet.

Composition of the experimental diet is detailed in Supplementary Table S1. Morphology (body weight and adiposity index) and food intake (energy intake and vitamin D intake) parameters were also controlled and measured (Supplementary Tables S2 and S3, respectively). Further details can be found in Bonnet et al. (2019) [11]. 

### 2.7. Plasma Sample Preparation

At the end of the 11-week diet period, the mice were fasted overnight and blood was collected by cardiac puncture under anesthesia. Plasma was obtained after centrifugation at 3000× *g* for 15 min at 4 °C, and stored at −80 °C. The plasma preparation procedure was inspired by Wang et al. [27]. As vitamin D and its metabolites are light-sensitive, the extraction procedure was conducted under low light. After thawing on ice, mouse plasma was centrifuged at 13,600× *g* for 15 min at 4 °C, then 100 µL of each sample was transferred to a glass tube with 10 µL of deuterated working solution. Proteins were precipitated by adding 500 µL of AcN, vortex-mixed, and centrifuged at 3000× *g* for 10 min at 4 °C. The supernatant was transferred to another glass tube, then the volume was reduced to one half under a nitrogen stream, and 500 µL of EtOAc was added to the solution for liquid-liquid extraction. After shaking vigorously for 10 min, samples were centrifuged at 590× *g* for 20 min at 4 °C, and the upper organic layer was transferred to a glass tube and reduced under nitrogen stream. The plasma samples were then derivatized with Amplifex^®^ Diene reagent.

For validation, a set of plasmas were pooled together and treated as described above.

### 2.8. Adipose Tissue Preparation

The animals were sacrificed by cervical dislocation under anesthesia, and the epididymal white adipose tissue (eWAT) was collected, weighed, snap-frozen in liquid nitrogen, and stored at −80 °C. The adipose tissue preparation procedure was inspired by Lipkie et al. [24]. After thawing on ice, 50 mg of tissue was ground with 1 mL of PBS at 30 mvt/s or 15 min (Retsch, Eragny-sur-Oise, France). Tissue homogenate was transferred into a glass tube and spiked with 25 µL of deuterated stock solution. AcN (1 mL) was added, and the mixture was vortexed for 5 min then centrifuged at 6000× *g* for 5 min at 4 °C. After addition of 1 mL of MTBE, the mixture was vortexed for 5 min, then centrifuged at 6000× *g* for 5 min at 4 °C, and the upper organic layer was collected in another tube. The same extraction was repeated twice more with additions of 300 µL of PBS, 1 mL of AcN (vortex + centrifugation) and 1 mL of MTBE (vortex + centrifugation). The supernatants were pooled then dried under nitrogen. Oasis HLB cartridges were prepared with 3 mL each of EtOAc, MeOH, and H_2_O. The samples were reconstituted with 1 mL of MeOH and 1 mL of K_2_HPO_4_ (0.4 M) and loaded on the cartridge. The cartridge was washed with 3 mL of H_2_O and 2 mL of 70% MeOH before being dried for 2 min under vacuum. Tips were washed with AcN and analytes were eluted with 1.5 mL of AcN and dried under nitrogen. Samples were then derivatized with Amplifex^®^ reagent.

For validation, a set of adipose tissues were pooled together and treated as described above.

### 2.9. Amplifex Derivatization

A one-step derivatization using Amplifex^®^ Diene as reagent was employed to improve the ionization efficiency of the metabolites [17]. The dried sample was added with 30 μL of Amplifex^®^ solution, vortexed for 15 s, then incubated for 30 min at room temperature. Next, 30 μL of deionized water was added to the sample, vortexed for 15 s, and transferred into vials for LC injection.

### 2.10. Method Validation

Our method was validated according to FDA criteria adapted from Shah et al. [28], and we evaluated specificity, selectivity, linearity, sensitivity, accuracy, precision, stability, and dilution tests.

#### 2.10.1. Linearity and Limits of Quantification (LoQ)

In order to assess the linearity of the quantification methods, we analyzed increasing amounts of analytical-standard solution spiked with the deuterated pool of the three corresponding analytes. For each derivatization reagent and each analyte, i.e., cholecalciferol, 25(OH)D3 and 1,25(OH)2D3, calibration curves were plotted with peak area ratio of vitamin D metabolite to the respective internal standard versus a range of analyte concentrations. For cholecalciferol, these two peaks represent diastereomers 13.2- and 13.5-min retention times. As the tandem-mass spectra showed the same pattern (same product ion and relative intensities), we summed their peak areas for the quantification.

Five concentrations of standards were chosen to cover the expected concentration range in the study. The series of concentrations were 0.20–50 ng/mL for cholecalciferol, 0.10–12.5 ng/mL for 25(OH)D3, and 0.020–2.5 ng/mL for 1,25(OH)2D3. We processed five replicates for each concentration level.

The LoQ was set as the concentration at which precision was less than 20% of the relative standard deviation, with an accuracy of between 80% and 120% of the theoretical value [29].

#### 2.10.2. Accuracy and Precision 

Replicated samples at three concentration levels were analyzed in separate runs to determine intra-day and inter-day precision and accuracy.

Intra-day accuracy and precision were calculated by processing five replicates at three concentration levels. Inter-day accuracy and precision were determined by analyzing five replicate samples in 3 batches (*n* = 15) at three concentration levels.

Precision of the assay was estimated by the coefficient of variation for each concentration level. Accuracy was represented as the relative error to the nominal concentrations (RE %), calculated as ratio of the deviation to the theoretical value ×100.

## 3. Statistical Analysis

The data are reported as means ± SEM. Significant differences were determined using a Student’s *t*-test, with the threshold for statistical significance set at *p* < 0.05.

## 4. Results and Discussion

### 4.1. Method Validation

#### 4.1.1. LC–HRMS/MS 

**Figure 1:**
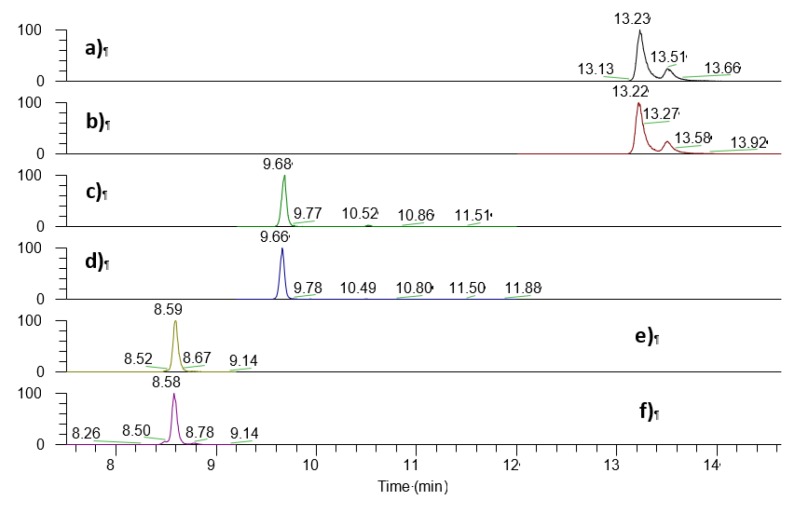
Extracted Ion Chromatography of standards mixed solution derivatized with Amplifex reagent. (**a**) *m*/*z* 657.43 extracted from the MS/MS spectrum of the *m*/*z* 716.50 ion of cholecalciferol, (**b**) *m*/*z* 660.45 extracted from the MS/MS spectrum of the *m*/*z* 719.50 ion of d3-cholecalciferol, (**c**) *m*/*z* 673.43 extracted from MS/MS spectrum of the *m*/*z* 732.51 ion of 25(OH)D3, (**d**) *m*/*z* 676.45 extracted from MS/MS spectrum of the *m*/*z* 735.50 ion of d3-25(OH)D3, (**e**) *m*/*z* 689.42 extracted from MS/MS spectrum of the *m*/*z* 748.51 ion of 1,25(OH)2D3 and **(f)** *m*/*z* 692.44 extracted from MS/MS spectrum of the *m*/*z* 751.50 ion of d3-1,25(OH)2D3.

The optimal separation was achieved with linear gradient conditions using AcN/H_2_O (with 0.1% FA) as mobile phase on a C18 column, as shown in Figure 1. 

The three vitamin D-Amplifex^®^ derivates were chromatographically resolved from each other (Peak Resolution: Rs >1.5). Tandem mass spectrometry analysis combined with satisfactory chromatographic separation ensured high specificity and selectivity of the method (Supplementary Figures S1 and S2).

The recently-developed reagent Amplifex is a 4-phenyl-1,2,4-triazoline-3,5-dione (PTAD) base with a naturally ionized quaternary ammonium group. It further enhances ionization efficiency compared to PTAD and thus achieves lower detection limits. This may explain the higher sensitivity of the Multiple Reaction Monitoring (MRM) methods for quantification of Amplifex-derivatized VD compared to PTAD-derivatized VD. As expected, the Amplifex reagent gave a lower LoQ than PTAD and thus emerges as the preferred method for derivatization. The scope of LC-MS/MS methods published to date has been limited to the quantification of one or two vitamin D metabolites in a single run. There is now a consensus that derivatization techniques can maximize both selectivity and sensitivity, and here we show the value of this derivatization method for enabling simultaneous and accurate LC-HRMS/MS quantification of cholecalciferol, 25(OH)D3, and 1,25(OH)2D3 in plasma and adipose tissue [16,17,19,20].

#### 4.1.2. Linearity, LoQ and Calibration Curves

The data were fitted using linear regression. Table 2 reports linear range and LoQ for the derivatized metabolites. Regression analysis showed good linearity with correlation coefficients (*r*^2^) >0.993 for the three analytes.

#### 4.1.3. Precision and Accuracy

Table 3 summarizes the precision (%CV) and accuracy (%RE) of the method according to derivatization procedure for simultaneous quantification of all three vitamin D metabolites. Using Amplifex^®^ derivatization, the intra-assay variation ranged between 0.8% and 6% depending on the vitamin D metabolite and concentration considered, and the inter-assay variation was between 2% and 14.4%. The RE of the three tested concentrations in three independent assays were within ±3%, ±9% and ±14% for cholecalciferol, 25(OH)D3 and 1,25(OH)2D3, respectively. These results indicate good reproducibility and accuracy of the method. The %CV and %RE are all <15%.

#### 4.1.4. Dilution Test and Stability 

The dilution test with high-concentration overloaded plasma samples showed between 83% and 110% recovery (data not shown).

We tested the stability of derivatized vitamin D metabolites. The Amplifex-derivatized metabolites showed a signal decrease after 24 h in the autosampler (5 °C) from −15.3 ± 0.5%, −19.7 ± 2.0%, −42.6 ± 2.0% for cholecalciferol, 25(OH)D3 and 1,25(OH)2D3, respectively (Data not shown). These stability results fit with what we expected based on Amplifex^®^ Diene reagent guidance for users.

### 4.2. Impact of High-Fat Diet on Vitamin D Metabolites in Plasma and in Adipose Tissue

After validation of the method, we quantified the three vitamin D metabolites in plasma and adipose tissue from the mouse study (Supplementary Figure S3).

In plasma, after 11 weeks of HF diet, compared to control diet, cholecalciferol concentration significantly decreased whereas 25(OH)D3 concentration increased and 1,25(OH)2D3 concentration remained unchanged. In eWAT, HF diet decreased cholecalciferol and 1,25(OH)2D3 concentrations but had no effect on 25(OH)D3 concentrations (Table 4). However, 25(OH)D3 was stronger in quantity terms (3.2-fold increase). These results are in agreement with Carrelli et al. [30], supporting the hypothesis that the large amount of adipose tissue in obese individuals serves as a reservoir for vitamin D. Here, our findings push the hypothesis further, with the quantification of 1,25(OH)2D3 concentration pointing to an active role of adipose tissue in the modulation of vitamin D metabolism during obesity. Note too that 1,25(OH)2D3 was also higher in quantity in obese mice compared to control mice (Table 4). 

## 5. Conclusions

The use of a recently developed derivatization reagent Amplifex^®^ affords dramatically enhanced ionization efficiency, thus achieving lower detection limits and making it possible to detect picomolar-concentration metabolites. We have developed and validated a powerful LC-HRMS/MS technique to simultaneously quantify key analytes of interest in complex samples other than plasma. Here we used the method to quantify 1,25(OH)2D3 along with cholecalciferol and 25(OH)D3 in adipose tissue to extend research into the hypothesis of vitamin D metabolite storage and production, especially in obesity settings [11].

## Figures and Tables

**Table 1:** Precursor and product ions *m*/*z* values, retention times, and normalized collision energies for Amplifex-derivatized cholecalciferol, 25(OH)D3, 1,25(OH)2D3, and the corresponding isotopically-labeled molecule for absolute quantification.

Compound Name	Precursor Ion	Precursor *m*/*z*	Product *m*/*z*	Retention Time (min)	Collision Energy (%)
Cholecalciferol-Amplifex	[M]+	716.50	657.43	13.2 + 13.5	21
25(OH)D3-Amplifex	[M]+	732.51	673.43	9.7	21
1,25(OH)2D3-Amplifex	[M]+	748.51	689.42	8.6	21
d3-cholecalciferol-Amplifex	[M]+	719.50	660.45	13.2 + 13.5	21
d3-25(OH)D3-Amplifex	[M]+	735.50	676.45	9.7	21
d3-1,25(OH)2D3-Amplifex	[M]+	751.50	692.44	8.6	21

**Table 2:** Linear range, limits of quantification (LoQ) and RE % for the 3 metabolites derivatized with Amplifex^®^. RE was determined for LoQ concentration (RE %: Relative error to the nominal concentrations).

Analytes	Linear Range ng/mL	LoQ ng/mL	RE %
Cholecalciferol	0.20–50	0.78	−5.3
25(OH)D3	0.10–12.5	0.19	4.0
1,25(OH)2D3	0.02–2.5	0.02	17.3

The range of linearity range was considered as the working range.

**Table 3:** Precision and accuracy of cholecalciferol, 25(OH)D3 and 1,25(OH)2D3 measurements.

Compound and Concentration	Intra-Assay (*n* = 5) ^a^			Inter-assay (*n* = 3) ^b^		
	Measured ^c^	%RE ^d^	%CV ^e^	Measured ^c^	%RE ^d^	%CV ^e^
Cholecalciferol						
1.56 ng/mL	1.53 ± 0.01	−1.7	1.0	1.57 ± 0.09	0.9	12.7
3.13 ng/mL	3.31 ± 0.06	6.2	2.6	3.09 ± 0.28	−1.2	9.0
12.5 ng/mL	12.9 ± 0.13	3.2	1.3	12.1 ± 0.25	−2.9	2.0
25(OH)D3						
0.78 ng/mL	0.81 ± 0.03	4.1	6.0	0.74 ± 0.09	9.3	11.1
1.56 ng/mL	1.70 ± 0.04	9.0	3.7	1.49 ± 0.10	−4.2	7.1
6.25 ng/mL	6.36 ± 0.12	1.8	2.7	5.83 ± 0.18	−6.8	3.1
1,25(OH)2D3						
0.16 ng/mL	0.15 ± 0.001	−6.3	1.5	0.18 ± 0.01	14.4	13.0
0.31 ng/mL	0.32 ± 0.002	5.1	0.8	0.31 ± 0.04	0.1	14.4
1.25 ng/mL	1.32 ± 0.02	5.8	1.9	1.23 ± 0.03	−1.6	2.8

^a^ intra-assay precision was calculated for 5 replicates measured in a single run; ^b^ inter-assay precision was calculated from 3 independent assays. ^c^ Means ± SD of measured concentrations. ^d^ Percent of relative error (%RE) is defined as the ratio of the deviation to the theoretical value ×100. ^e^ Coefficient of variance is defined as the ratio of the standard deviation to the mean value ×100.

**Table 4:** Concentrations and quantities of the three Amplifex-derivatized vitamin D_3_ metabolites in mouse plasma and adipose tissue after 11 weeks of diet. The quantities in adipose tissue were calculated as concentration x mass of epididymal white adipose tissue (eWAT).

	Cholecalciferol	25(OH)D3	1,25(OH)2D3
Plasma concentration			
Control	2.6 ± 0.1 ng/mL	38.4 ± 0.9 ng/mL	0.24 ± 0.02 ng/mL
HF	1.3 ± 0.1 ng/mL *	42.7 ± 1.4 ng/mL *	0.24 ± 0.01 ng/mL
Adipose tissue concentration			
Control	332 ± 25 ng/g	51 ± 4 ng/g	45 ± 6 ng/g
HF	236 ± 31 ng/g *	62 ± 8 ng/g	26 ± 6 ng/g *
Adipose tissue quantity			
Control	284 ± 29 ng	44 ± 5 ng	40 ± 6 ng
HF	548 ± 61 ng *	140 ± 12 ng *	61 ± 14 ng *

Control diet: *n* = 10, HF diet, high-fat diet: *n* = 10. Values are presented as means ± SEM. Significant differences were determined by a Student’s *t*-test * *p* < 0.05.

## Supplementary Materials

The following are available online at https://www.mdpi.com/2072-6643/11/9/1977/s1, Figure S1: MS/MS spectrum of 1,25(OH)2D3, 25(OH)D3 and cholecalciferol derivatized with Amplifex reagent and their respective deuterated forms, Figure S2: Derivatization reaction, Figure S3: Extrated Ion Chromatography of plasma (**A**) and adipose tissue (**B**) sample derivatized with Amplifex reagent (a) *m*/*z* 657.43 extracted from the MS/MS spectrum of *m*/*z* 716.50 ion of cholecalciferol-Amplifex, (b) *m*/*z* 660.45 extracted from the MS/MS spectrum of *m*/*z* 719.50 ion of d3-cholecalciferol-Amplifex, (c) *m*/*z* 673.43 extracted from MS/MS spectrum of *m*/*z* 732.51 ion of 25(OH)D3-Amplifex, (d) *m*/*z* 676.45 extracted from MS/MS spectrum of *m*/*z* 735.50 ion of d3-25(OH)D3-Amplifex, (e) *m*/*z* 689.43 extracted from MS/MS spectrum of *m*/*z* 748.51 ion of 1,25(OH)2D3-Amplifex and (f) *m*/*z* 692.44 extracted from MS/MS spectrum of *m*/*z* 751.50 ion of d3-1,25(OH)2D3-Amplifex, Table S1: Experimental diet compositions, Table S2: Mice morphologic parameters, Table S3: Food intake parameters.
