# Supplementary material for: Simple Fast Quantification of Cholecalciferol, 25-Hydroxyvitamin D and 1,25-Dihydroxyvitamin D in Adipose Tissue Using LC-HRMS/MS
Source: Nutrients. 2019 Aug 22;11(9):1977. doi: 10.3390/nu11091977 (PMC6770531; doi:10.3390/nu11091977)
Supplement: Supplementary file 1 [file nutrients-11-01977-s001.pdf]

Supplementary

Table 1. Experimental diet compositions.

Item (g)	Control diet	High fat diet
**Sucrose**	33.13	8.85
**Dextrin**	29.86	0
**Casein—Vitamin, Tested**	18.96	25.85
**Powdered Cellulose**	4.74	6.41
**Maltodextrin**	3.32	16.15
**Soybean oil**	2.37	3.23
**Lard**	1.90	31.66
**Potassium Citrate, Tribasic Monohydrate**	1.56	2.13
**Calcium Phosphate**	1.23	1.68
**DIO Mineral Mix**	0.95	1.29
**AIN-76A Vitamin mic**	0.95	1.29
**Calcium Carbonate**	0.52	0.71
**L-Cystine**	0.28	0.39
**Choline Bitartrate**	0.19	0.26
**FD&C Yellow 5 Lake**	0.05	0.05
**Total**	100	100

Table 2. Mice morphologic parameters.

	Control	HF
**Body weight at start (g)**	22.4 ± 0.2	22.2 ± 0.1
**Body weight at the end (g)**	31.5 ± 0.5	42.2 ± 1.3 *
**Adiposity index**	4.9 ± 1.4	10.3 ± 0.9 *

Values are presented as means ± SEM. Student's t-test was used, p values: *. $p < 0.05$ between control group and high fat group (HF) for 11 weeks.

Table 3. Food intake parameters.

	Energy intake (kcal/g)	Vitamin D intake (UI/g/day)
**Control**	14.4 ± 0.8	3.4 ± 0.2
**HF**	14.2 ± 0.5*	3.6 ± 0.2 *

Values are presented as means ± SEM. Student's t-test was used, p values: *. $p < 0.05$ between control group and high fat group (HF) for 11 weeks.

1,25(OH)$_2$D$_3$-Amplifex and d3-1,25(OH)$_2$D$_3$-Amplifex

25(OH) D$_3$-Amplifex and d3-25(OH)D$_3$-Amplifex

Cholécalciferol-Amplifex and d3-Cholécalciferol-Amplifex

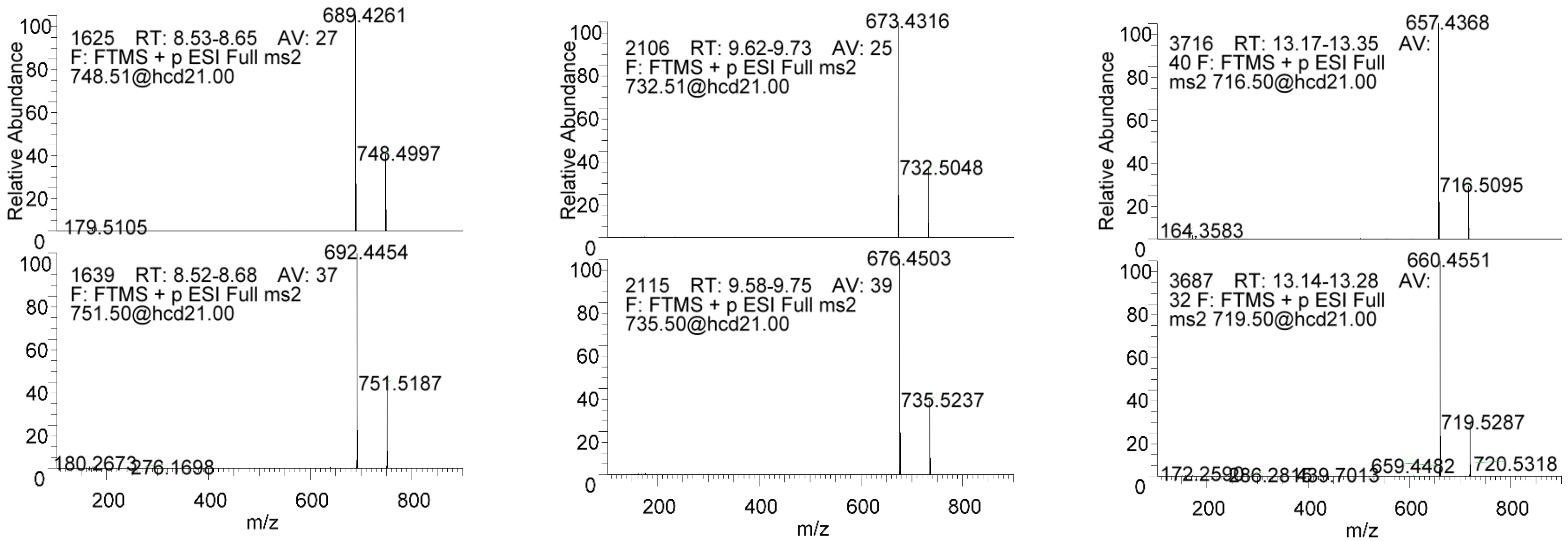

Figure 1. MS/MS spectrum of 1,25(OH)$_2$D$_3$, 25(OH)D$_3$ and cholecalciferol derivatized with Amplifex reagent and their respective deuterated forms.

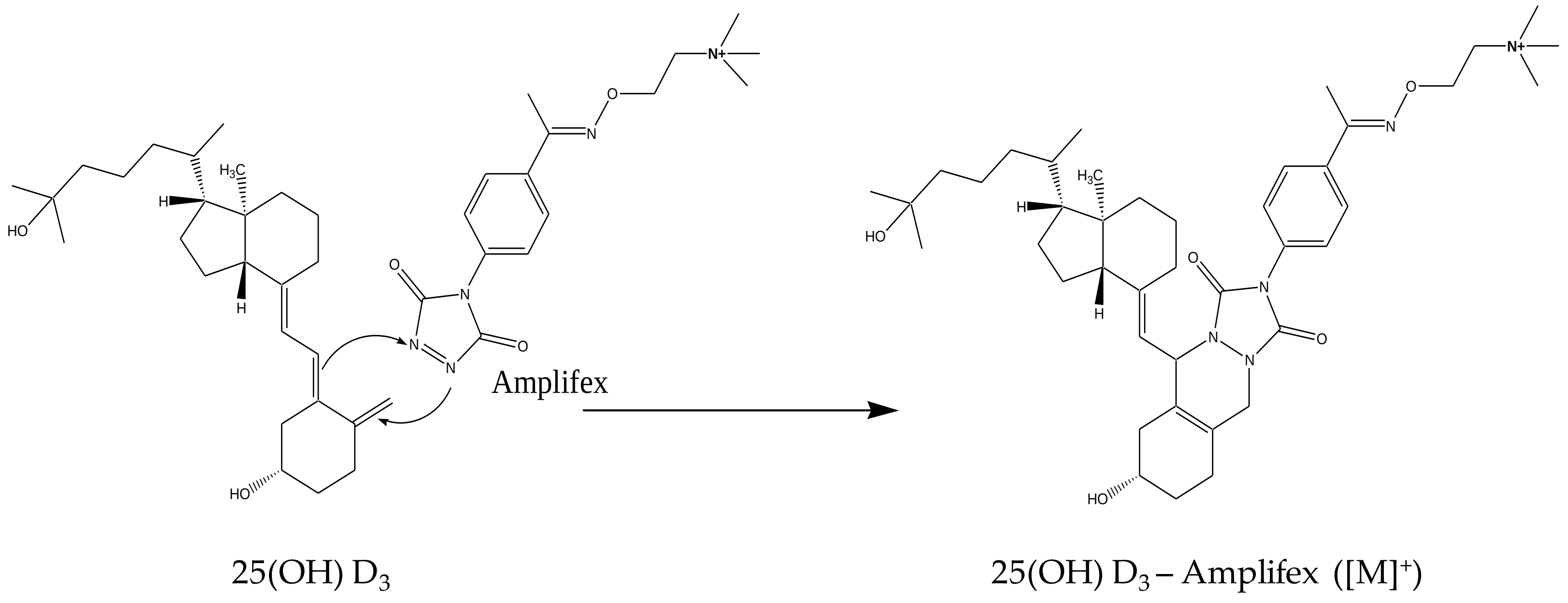

Figure 2. Derivatization reaction.

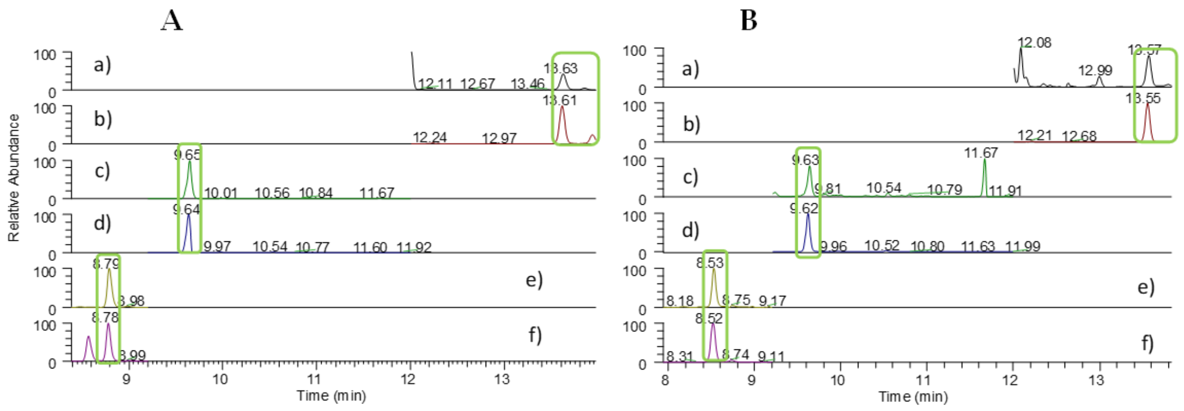

Figure 3. Extracted Ion Chromatography of plsma (**A**) and adipose tissue (**B**) sample derivatized with Amplifex reagent (a) m/z 657.43 extracted from the MS/MS spectrum of m/z 716.50 ion of cholecalciferol-Amplifex, (b) m/z 660.45 extracted from the MS/MS spectrum of m/z 719.50 ion of d3-cholecalciferol-Amplifex, (c) m/z 673.43 extracted from MS/MS spectrum of m/z 732.51 ion of 25(OH)D_3-Amplifex, (d) m/z 676.45 extracted from MS/MS spectrum of m/z 735.50 ion of d3-25(OH)D_3-Amplifex, (e) m/z 689.43 extracted from MS/MS spectrum of m/z 748.51 ion of 1,25$(OH)_2$D3-Amplifex and (f) m/z 692.44 extracted from MS/MS spectrum of m/z 751.50 ion of d3-1,25$(OH)_2$D3-Amplifex.
